# Supplementary material for: Decreased quality of life and treatment satisfaction in patients with latent autoimmune diabetes of the adult
Source: PeerJ. 2017 Oct 18;5:e3928. doi: 10.7717/peerj.3928 (PMC5650726; doi:10.7717/peerj.3928)
Supplement: File S1 [file peerj-05-3928-s001.pdf]

ADDQoL

Este cuestionario pregunta sobre su calidad de vida – en otras palabras, qué tan buena o mala siente que es su vida.

Por favor, ponga una “X” en el cuadrito que mejor describa su respuesta para cada pregunta.

Lo que queremos saber es cómo se siente con su vida actualmente.

I) En general, mi calidad de vida actual es:

☐	☐	☐	☐	☐	☐	☐
excelente	muy buena	buena	ni buena ni mala	mala	muy mala	extrema-damente mala

Ahora nos gustaría saber cómo su calidad de vida es afectada por la diabetes, su manejo (incluyendo medicamentos, visitas al médico y comida) y cualquier complicación que pueda tener.

II) Si yo *no* tuviera diabetes, mi calidad de vida sería:

☐	☐	☐	☐	☐
muchísimo mejor	mucho mejor	un poco mejor	la misma	peor

For information only

This copy is for information only - for use, please contact Professor Bradley
ADDQoL © Prof Clare Bradley: 24.2.94. Spanish for USA 26.7.07 (from standard UK English rev. 1.3.06)

Health Psychology Research, Dept of Psychology, Royal Holloway, University of London, Egham, Surrey, TW20 0EX, UK

Por favor, responda a las preguntas más específicas en las páginas siguientes. Para cada aspecto de vida descrito, encontrará dos partes:

Para la parte (a):	ponga una "X" en el cuadrito para demostrar cómo la diabetes afecta este aspecto de su vida;
Para la parte (b):	ponga una "X" en el cuadrito para indicar lo importante que es este aspecto de la vida en su calidad de vida.

For information only

1	(a)	**Si yo *no* tuviera diabetes, disfrutaría de mis actividades de tiempo libre:**				
		☐ muchísimo más	☐ mucho más	☐ un poco más	☐ igual	☐ menos
	(b)	**Mis actividades de tiempo libre son:**				
		☐ muy importantes	☐ importantes	☐ algo importantes	☐ nada importantes	

2		**¿Está usted trabajando actualmente, buscando trabajo o le gustaría trabajar?**				
		**Sí** ☐ Si la respuesta es ***sí***, complete **(a)** y **(b)**.				
		**No** ☐ Si la respuesta es ***no***, pase directamente a la **Pregunta 3**.				
	(a)	**Si yo *no* tuviera diabetes, mi vida de trabajo sería:**				
		☐ muchísimo mejor	☐ mucho mejor	☐ un poco mejor	☐ igual	☐ peor
	(b)	**Para mí, tener una vida de trabajo es:**				
		☐ muy importante	☐ importante	☐ algo importante	☐ nada importante	

3	(a)	**Si yo *no* tuviera diabetes, los viajes cortos o largos serían:**				
		☐ muchísimo más fáciles	☐ mucho más fáciles	☐ un poco más fáciles	☐ iguales	☐ más difíciles
	(b)	**Para mí, los viajes cortos o largos son:**				
		☐ muy importantes	☐ importantes	☐ algo importantes	☐ nada importantes	

.

This copy is for information only - for use, please contact Professor Bradley
