# Supplementary material for: Decreased quality of life and treatment satisfaction in patients with latent autoimmune diabetes of the adult
Source: PeerJ. 2017 Oct 18;5:e3928. doi: 10.7717/peerj.3928 (PMC5650726; doi:10.7717/peerj.3928)
Supplement: File S2 [file peerj-05-3928-s002.pdf]

Cuestionario de Satisfacción con el Tratamiento para la Diabetes: DTSQs

Las siguientes preguntas están relacionadas con el tratamiento de su diabetes (incluyendo insulina, comprimidos y/o dieta) y su experiencia en estas últimas semanas. Por favor conteste a cada pregunta haciendo un círculo en un número de cada una de las escalas.

1. ¿En qué medida está Vd. satisfecho/a con su tratamiento actual?

muy satisfecho/a	6	5	4	3	2	1	0	muy insatisfecho/a

2. Últimamente, ¿con qué frecuencia ha considerado que su nivel de azúcar en la sangre era inaceptablemente alto?

la mayoría del tiempo	6	5	4	3	2	1	0	nunca

For information only

This copy is for information only - for use, please contact Professor Bradley
DTSQs © Prof Clare Bradley 9/93. Spanish for Spain 30.4.03 (from standard UK English rev. 7/94)
Health Psychology Research, Dept of Psychology, Royal Holloway, University of London, Egham, Surrey, TW20 0EX, UK.
