# Supplementary material for: Decreased quality of life and treatment satisfaction in patients with latent autoimmune diabetes of the adult
Source: PeerJ. 2017 Oct 18;5:e3928. doi: 10.7717/peerj.3928 (PMC5650726; doi:10.7717/peerj.3928)
Supplement: Table S1 [file peerj-05-3928-s003.docx]

**Table S1.** Estimate difference in QoL based on model in Table 4 for the interaction between group, insulin treatment and diabetic retinopathy for the Audit of Diabetes Dependent Quality of Life (ADDQoL)

| Reference group | LADA * insulin * no DR | | | LADA * insulin * DR | | |
| --- | --- | --- | --- | --- | --- | --- |
| Coefficients | Estimate | Standard  error | p value | Estimate | Standard  error | p value |
| LADA * insulin * DR | -1.25952 | 0.40942 | 0.002 | - | - | - |
| LADA * insulin * no DR | - | - | - | 1.25952 | 0.40942 | 0.002 |
| T2DM * insulin * no DR | 0.10006 | 0.31884 | 0.750 | 1.35958 | 0.45169 | 0.003 |
| T1DM * no DR | -0.04103 | 0.22328 | 0.850 | 1.21849 | 0.37931 | 0.001 |
| T2DM * insulin * DR | 0.07964 | 0.22639 | 0.730 | 1.33916 | 0.37462 | <0.001 |
| T1DM * DR | -0.31735 | 0.26548 | 0.230 | 0.94217 | 0.38658 | 0.020 |
| LADA * without insulin * no DR | 1.06176 | 0.64037 | 0.100 | 2.32128 | 0.71636 | 0.001 |
| T2DM * without insulin * no DR | 0.95200 | 0.20699 | <0.001 | 2.21152 | 0.38198 | <0.001 |
| LADA * without insulin * DR | 0.90845 | 0.77258 | 0.240 | 2.16797 | 0.83688 | 0.010 |
| T2DM * without insulin * DR | 0.62161 | 0.22575 | 0.006 | 1.88112 | 0.39198 | <0.001 |

*stand for the existence of interactions between variables.

LADA : latent autoimmune diabetes of adult, T2DM: type 2 diabetes mellitus, T1DM: type 1 diabetes mellitus, DR: diabetic retinopathy
