# Supplementary material for: Decreased quality of life and treatment satisfaction in patients with latent autoimmune diabetes of the adult
Source: PeerJ. 2017 Oct 18;5:e3928. doi: 10.7717/peerj.3928 (PMC5650726; doi:10.7717/peerj.3928)
Supplement: Table S2 [file peerj-05-3928-s004.docx]

**Table S2.** Multivariate linear regression for the Diabetes Treatment Satisfaction Questionnaire (DTSQ) final score

| Coefficients | Estimate | Standard error | p value |
| --- | --- | --- | --- |
| Intercept | 24.3100 | 1.0204 | <0.001 |
| T2DM * without insulin | 1.8284 | 0.9904 | 0.070 |
| LADA * without insulin | 1.1482 | 2.7750 | 0.680 |
| T2DM * insulin | -0.8961 | 1.0759 | 0.410 |
| T1DM | 1.6721 | 1.0374 | 0.110 |
| Physical activity | 1.8967 | 0.5770 | 0.001 |
| Smoker, current | 0.5643 | 0.7011 | 0.420 |
| Smoker, former | -1.5546 | 0.6340 | 0.010 |

Multiple R-squared: 0.07293 (5 cases with missing information for any variable in the model).

Reference group: LADA patients with insulin treatment.

*stand for the existence of interactions between variables.

LADA: latent autoimmune diabetes of adult, T2DM: type 2 diabetes mellitus, T1DM: type 1 diabetes mellitus.
