# Supplementary material for: Decreased quality of life and treatment satisfaction in patients with latent autoimmune diabetes of the adult
Source: PeerJ. 2017 Oct 18;5:e3928. doi: 10.7717/peerj.3928 (PMC5650726; doi:10.7717/peerj.3928)
Supplement: Table S3 [file peerj-05-3928-s005.docx]

**Table S3.** Multivariate linear regression for Diabetes Treatment Satisfaction Questionnaire (DTSQ) hyperglycaemia frequency perception

| Coefficients | Estimate | Standard error | p value |
| --- | --- | --- | --- |
| Intercept | 0.10531 | 0.61917 | 0.870 |
| T2DM * without insulin | -1.17472 | 0.30468 | <0.001 |
| LADA * without insulin | -0.37881 | 0.85188 | 0.660 |
| T2DM * insulin | -0.68052 | 0.35174 | 0.050 |
| T1DM | -0.66661 | 0.32005 | 0.040 |
| HbA1c | 0.52855 | 0.07155 | <0.001 |
| Physical activity | -0.55232 | 0.17756 | 0.002 |
| DR | 0.36839 | 0.18777 | 0.050 |

Multiple R-squared: 0.1991 (7 cases with missing information for any variable in the model).

Reference group: LADA patients with insulin treatment.

*stand for the existence of interactions between variables.

LADA: latent autoimmune diabetes of adult, T2DM: type 2 diabetes mellitus, T1DM: type 1 diabetes mellitus, HbA1c: glycated haemoglobin.
