# Supplementary material for: Decreased quality of life and treatment satisfaction in patients with latent autoimmune diabetes of the adult
Source: PeerJ. 2017 Oct 18;5:e3928. doi: 10.7717/peerj.3928 (PMC5650726; doi:10.7717/peerj.3928)
Supplement: Table S4 [file peerj-05-3928-s006.docx]

**Table S4.** Multivariate linear regression for Diabetes Treatment Satisfaction Questionnaire (DTSQ) hypoglycaemia frequency perception

| Coefficients | Estimate | Standard error | p value |
| --- | --- | --- | --- |
| Intercept | 1.298113 | 0.290328 | <0.001 |
| T2DM * without insulin | -0.794643 | 0.288756 | 0.006 |
| LADA * without insulin | -0.507397 | 0.798883 | 0.530 |
| T2DM * insulin | 0.093348 | 0.312445 | 0.770 |
| T1DM | 0.699939 | 0.307130 | 0.020 |
| Female sex | 0.545123 | 0.159371 | 0.001 |
| Disease duration | 0.024874 | 0.009083 | 0.006 |

Multiple R-squared: 0.2165 (3 cases with missing information for any variable in the model).

Reference group: LADA patients with insulin treatment.

*stand for the existence of interactions between variables.

LADA: latent autoimmune diabetes of adult, T2DM: type 2 diabetes mellitus, T1DM: type 1 diabetes mellitus.
